# Smart Continence Care for People With Profound Intellectual and Multiple Disabilities: Protocol for a Cluster Randomized Trial and Trial-Based Economic Evaluation

**DOI:** 10.2196/42555

**Published:** 2022-11-22

**Authors:** Vivette J C van Cooten, Marieke F M Gielissen, Ghislaine A P G van Mastrigt, Wouter den Hollander, Silvia M A A Evers, Odile Smeets, Filip Smit, Brigitte Boon

**Affiliations:** 1 Academy Het Dorp, Research & Advisory on Technology in Long-term Care Arnhem Netherlands; 2 Tranzo Tilburg School of Social and Behavioral Sciences Tilburg University Tilburg Netherlands; 3 Siza, Center for Long-term Care for People with Disabilities Arnhem Netherlands; 4 Care and Public Health Research Institute (Research School CAPHRI) Maastricht University Maastricht Netherlands; 5 Department of Health Services Research Maastricht University Maastricht Netherlands; 6 Trimbos Institute, Netherlands Institute of Mental Health and Addiction Utrecht Netherlands; 7 Department of Epidemiology and Biostatistics and Department of Clinical Psychology Public Health Research Institute University Medical Centers Amsterdam Amsterdam Netherlands

**Keywords:** care technology, implementation, disability care, profound intellectual and multiple disabilities, economic evaluation, continence care, smart diaper

## Abstract

**Background:**

People with profound intellectual and multiple disabilities (PIMD) cannot communicate the need to change their incontinence products. The smart continence care (SCC) product Abena Nova signals caregivers when change is needed. This provides the opportunity for more person-centered care, increased quality of life, and a decreased number of leakages. However, there is a need for evidence of the effectiveness and cost-effectiveness of such technology compared with regular continence care (RCC) for people with PIMD.

**Objective:**

This paper presents the research protocol for an effectiveness and cost-effectiveness study with people with PIMD living in long-term care facilities in the Netherlands.

**Methods:**

A cluster randomized trial will be conducted in 3 consecutive waves across 6 long-term care providers for people with disabilities and 160 participants with PIMD. Long-term care providers are randomized at a 1:1 ratio, resulting in an intervention group and a group continuing RCC. The intervention group will receive implementation guidance and use SCC for 3 months; the other group will continue their RCC as usual and then switch to SCC. This study consists of three components: effectiveness study, economic evaluation, and process evaluation. The primary outcome will be a change in the number of leakages. The secondary outcomes are quality of life, the difference in the number of changes, the work perception of caregivers, cost-effectiveness, and cost utility. Data collection will occur at T0 (baseline), T1 (6 weeks), T2 (12 weeks), and T3 (9-month follow-up) for the first 2 intervention groups. An intention-to-treat analysis will be performed. The economic evaluation will be conducted alongside the trial from the societal and long-term care provider perspectives. Qualitative data collection through interviews and field notes will complement these quantitative results and provide input for the process evaluation.

**Results:**

This research was funded in December 2019 by ZonMw, the Netherlands Organization for Health Research and Development. As of June 2022, we enrolled 118 of the 160 participants. The enrollment of participants will continue in the third and fourth quarters of 2022.

**Conclusions:**

This study will provide insights into the effectiveness and cost-effectiveness of SCC for people with PIMD, allowing long-term care providers to make informed decisions about implementing such a technology. This is the first time that such a large-scale study is being conducted for people with PIMD.

**Trial Registration:**

ClinicalTrials.gov NCT05481840; https://clinicaltrials.gov/ct2/show/NCT05481840

**International Registered Report Identifier (IRRID):**

DERR1-10.2196/42555

## Introduction

### Background

People with profound intellectual and multiple disabilities (PIMD) depend entirely on professional care. Their disability is characterized by profound intellectual disability, that is, a developmental age of up to 24 to 36 months depending on the definition [1], profound motor disability, and, usually, secondary disabilities or impairments [2]. The Netherlands has approximately 9500 (April 1, 2013) people with severe intellectual disabilities or PIMD (developmental age of up to 4 years), of which 95% live in a long-term care facility [3].

People with PIMD commonly experience urinary and fecal incontinence. The percentage of incontinence ranges from 45% in people with severe intellectual disabilities [4] to 56% in people with PIMD. An incidence of 61% was reported in females with a specific form of PIMD: the Rett syndrome [5]. There is a correlation between an increased level of intellectual disability and a higher rate of incontinence [4,6] and between an increased level of physical impairment and a higher rate of incontinence [7]. There are methods to promote continence in people with PIMD, such as toilet routine training [4,8]. This training can take a long time and might not be successful for all persons with PIMD, as it requires a combination of communicative skills, mobility, and cognitive ability, skills which are commonly underdeveloped in people with PIMD [2]. In Dutch long-term care facilities, most people with PIMD who are incontinent wear pads, adult diapers, or catheters. When a person cannot notify when change is needed, the material is often changed at scheduled moments. However, these scheduled moments result in leakages when the material is oversaturated, leading to an additional change of clothing or bed sheets, and the person may need to be washed or showered.

Furthermore, long exposure to wet incontinence materials could result in skin problems, such as incontinence-associated dermatitis [9]. In addition, scheduled changes could result in unnecessary changes when the pads or diapers are still (relatively) dry. Leakages, skin problems, and unnecessary changes cause an extra burden to people with PIMD, resulting in agitation and additional transfers, and their caregivers, as unnecessary time is spent on continence care and related activities.

### Person-Centered Continence Care by Using Technology

The Health and Youth Care Inspectorate (Dutch Ministry of Health, Welfare, and Sports) emphasizes the importance of providing good care for people with PIMD, as they fully depend on their caregivers. The key point in providing good care to people with PIMD is to recognize the needs of the person and act accordingly, which is known as person-centered care [10,11]. However, it is complex to recognize these needs [12], and a caregiver must have known the person for many years [10]. The needs of people with PIMD regarding continence care can be communicated using technology (smart continence care [SCC]). Sensors in the incontinence material signal when a change of the material is needed. If the use of person-centered continence care decreases the number of leakages and unnecessary changes, it has the potential to increase the quality of care provided for people with PIMD, and it may also save the time spent on continence care and reduce the workload of the caregivers. This is even more important given the increasing shortage of health care workers [13], especially because PIMD care in the Netherlands has difficulties finding and keeping caregivers [14].

Several solutions have been developed to inform caregivers of when to change incontinence products. Some examples of such solutions are analog indicators on the material itself; a strip on the outside of the incontinence product that shifts color with changes in saturation [15]; smart continence products using sensor technology, such as a 72-hour observation of the voiding pattern registered by a small device attached to the incontinence product with an integrated sensor [16]; solutions for continuous monitoring and notification of the need for change with reusable sensors that are attached on the outside of the product [16]; and solutions with integrated sensors and removable clip [17]. To check the color change on the analog indicator, the caregiver should physically inspect the product. With the 72-hour observation technology, there is no real-time notification of the need for change. Both can be considered disadvantages. With the last 2 solutions, caregivers and people with PIMD can benefit from real-time notification sent to a mobile phone when a change of continence material is needed. This study investigates a smart continence product with integrated sensors and a removable clip (Abena Nova).

Previous studies on SCC, mostly pilot studies, have investigated its effect on the number of leakages, number of changes, and quality of life of the user [18,19]. However, these studies were small and had different target groups (ie, older people and people with intellectual disabilities), and in one of the studies [18], the supplier was involved in the research. Therefore, there is a need for independent and comprehensive research.

### Aim of the Study

This paper presents the research protocol (according to the SPIRIT [Standard Protocol Items: Recommendations for Interventional Trials] guideline [20]) for the “Smart Diaper research and implementation project” to evaluate the effectiveness, cost-effectiveness, and implementation process of SCC for people with PIMD living in a long-term care facility. Thorough research on the effects, added value, and costs will help the funding bodies and policy makers of long-term care facilities to make an informed decision about whether to implement smart continence products for people with PIMD.

Besides the societal and economic values of such a study, this research is unique from an academic perspective. Literature reviews have shown that the number of studies on the effectiveness of (technological) interventions for people with PIMD is very limited. Maes et al [21] revealed a total of 16 intervention studies between 1995 and 2006, and Dupont et al [22] showed a total of 39 studies (including several follow-up studies based on the same initial study, thus containing the same group of respondents) between 2006 and 2018. The reported sample size for most studies was relatively small; only 5 studies reported a sample size of >10 participants, and the largest study included 44 participants with PIMD. Likewise, economic evaluations of interventions for people with PIMD are rare [23]. Therefore, this first well-powered cluster randomized trial (CRT) aims to evaluate the effectiveness, cost-effectiveness, and implementation process of SCC for people with PIMD.

### Research Questions

This study will compare SCC with RCC provided to people with PIMD living in a long-term care facility in the Netherlands. This study consists of 3 parts, each with its focus and research questions. Textbox 1 lists the 3 parts and corresponding research questions.

**The 3 parts of this study and corresponding research questions.**

**Effectiveness study**

1. What is the effect of smart continence care (SCC) for people with profound intellectual and multiple disabilities (PIMD) on the number of leakages compared with regular continence care (RCC)?
2. What is the effect of SCC on the number of changes of incontinence material compared with RCC?
3. What is the effect of SCC on the quality of life of people with PIMD compared with RCC?
4. What is the effect of SCC on the work perception of caregivers with regard to continence care compared with RCC?

**Economic evaluation**

1. What is the cost-effectiveness of SCC compared with RCC provided to people with PIMD from a societal perspective?
2. What is the cost utility of SCC compared with RCC provided to people with PIMD?

**Process evaluation**

1. What are the experiences of the participating long-term care providers with respect to the implementation of SCC?

## Methods

### Study Design

This study design can be best described as a staggered-entry CRT. A total of 6 long-term care providers for people with disabilities are divided into 3 pairs. This allocation is done in consultation with the long-term care providers, depending on their readiness for the research and implementation of SCC. Within these pairs, the long-term care providers are randomized into the SCC or RCC conditions using random.org (1:1 ratio). The research started at the end of 2021 for the first pair, and the second and third pairs started during the first and second halves of 2022, respectively. The long-term care providers randomized into the RCC condition will implement SCC once data collection is completed (Table 1). Considering the complexity of successfully implementing health care technologies [24], time and effort are required to realize the full potential of SCC. With this design, a small team of researchers and implementation consultants can consecutively support all long-term care providers with the implementation of SCC, as it allows lessons learned to spill over to the second and third pairs. The intervention does not allow for blinding within the trial. Furthermore, the study has a mixed methods design to answer the research questions, using quantitative as well as qualitative data.

**Table 1:** Timeline within the staggered-entry cluster randomized trial.

			T0 (week 0)	Start SCC^a^		T1 (week 6)		T2 (week 12)	Start SCC		T3 (9 months)
**Couple 1 Q3-Q4 2021 and Q2 2022**											
	Long-term care provider A	✓		✓	✓		✓			✓	
	Long-term care provider B	✓			✓		✓		✓	—^b^	
**Couple 2 Q1-Q2 2022 and Q4 2022**											
	Long-term care provider C	✓		✓	✓		✓			✓	
	Long-term care provider D	✓			✓		✓		✓	—^b^	
**Couple 3 Q3-Q4 2022**											
	Long-term care provider E	✓		✓	✓		✓			N/A^c^	
	Long-term care provider F	✓			✓		✓		✓	N/A^c^	

^a^SCC: smart continence care.

^b^Will be based on data collected at T2.

^c^N/A: not applicable. This measurement cannot be completed in the allotted time frame.

### Ethics Approval

This study has been reviewed and approved by the Medical Ethics Committee of Radboudumc (NL72751.091.20). The trial has been registered at ClinicalTrials.gov (NCT05481840). Any modification to the study protocol will be checked with the funding body (outside of the annual progress update) and, if necessary, with the Medical Ethics Committee. The legal representatives of the participants will provide their written consent for the person with PIMD to participate in the research. Caregivers consent to participating and completing the web-based questionnaire by reading the information before the start and continuing to answer the questionnaire. The World Health Organization trial registration data set is presented in Multimedia Appendix 1.

### Sampling

#### Power

The power calculations for the study are based on the observed effect sizes in previous studies that used the same outcome measure. Bouman et al [18] and Nap et al [19] reported 73% and 62% reductions in the number of leakages, respectively. As these studies were fairly small, this study will be powered to detect a rather conservative reduction of 40%. By using 3 measurements (T0, T1, and T2) per participant across 6 clusters (each long-term care provider being one cluster), with an assumed intraclass correlation coefficient (ICC) of 0.01, an α value of .05, and a design effect of 1.05, the study will be adequately powered (80%) to detect an incidence rate ratio (IRR) of 0.60 (1-0.40) if 80 (50%) participants per arm are included (N=160). In addition, the power calculation is cross-checked hereto using a simulation procedure from the R package simstudy [25], in which each unique combination of ICCs (0.01, 0.05, and 0.10), IRRs (0.5, 0.6, 0.7, and 0.8), the number of clusters (6, 8, and 10), and the number of participants per cluster (8, 12, 16, 20, and 24) is used to generate 100 simulated data sets in which ρ=0.75 between consecutive time points. Each data set is subsequently analyzed using a generalized linear mixed model with a Poisson link function (R package lme4 [26]). The respective power of each combination of parameters is subsequently inferred from the fraction of the resulting *P* values <.05 of the treatment indicator (SCC vs RCC). This simulation shows that our study will be well powered (80%) to detect a 20% reduction (IRR=0.8) in the number of leakages when 6 clusters with 24 participants each are included, irrespective of the ICC. We aim to include 6 long-term care providers containing 27 participants each, thus resulting in 160 participants overall.

#### Selecting Long-term Care Providers

A total of 6 Dutch long-term care providers for people with disabilities were recruited before the grant proposal.

They should meet the following conditions:

- Provide residential care to people with PIMD and be able to provide at least 27 participants for the study
- Have an intention to implement SCC people with PIMD sustainably
- Have an IT infrastructure to support the use of SCC
- Show commitment to implementation and participation in the research by doing the following:
  - Signing an intention agreement at the level of management, middle management, and caregivers
  - Releasing funding for the purchase of the product
  - Providing human resources for implementation and training
  - Providing a project leader to coordinate the project

Long-term care providers will receive a financial contribution for the research activities, up to a maximum amount of €10,000 (US $9816.50, currency rate as per October 19, 2022), when different targets are met (such as enrolling 27 participants and completing data collection) to promote full participation in the Smart Diaper research and implementation project.

#### Participant Selection

A long-term care provider for disabled people often has multiple locations (eg, “houses” or “residential groups”) where people with disabilities live. Most of these locations have permanent teams of caregivers. When selecting locations with people with PIMD, favorable conditions for implementation will be checked, such as willingness to implement SCC; low staff turnover; and whether other priorities could jeopardize the research and implementation, such as other (research) projects, renovation, or relocation. This is important because research and implementation will require commitment, resources, and time. The locations of each long-term care provider are recruited after the randomization. Information on the study sites will be available upon request from the corresponding author.

After locations are selected, individual participants will be eligible for inclusion in the study if they are aged >18 years, have PIMD, use incontinence products, and are not able to communicate the need for a change of the incontinence material, and their legal representatives will give informed consent for them to participate in the study. Participants who use a permanent catheter or show behavior that may result in a risk for the patient when using SCC (such as pica disorder) are excluded from the study. There are 2 possible soft exclusion criteria in which participants should be carefully considered: release of feces ≥3 times per 24 hours, as this may interfere with the technology detecting urine and a behavior that can hinder the successful implementation of SCC, such as, but not limited to, removing the incontinence material, clip, or clothing. Caregivers will be encouraged to make a thoughtful decision in this situation regarding whether it is meaningful to try SCC for these people and how these potential impediments can be mitigated.

**Figure 1:**
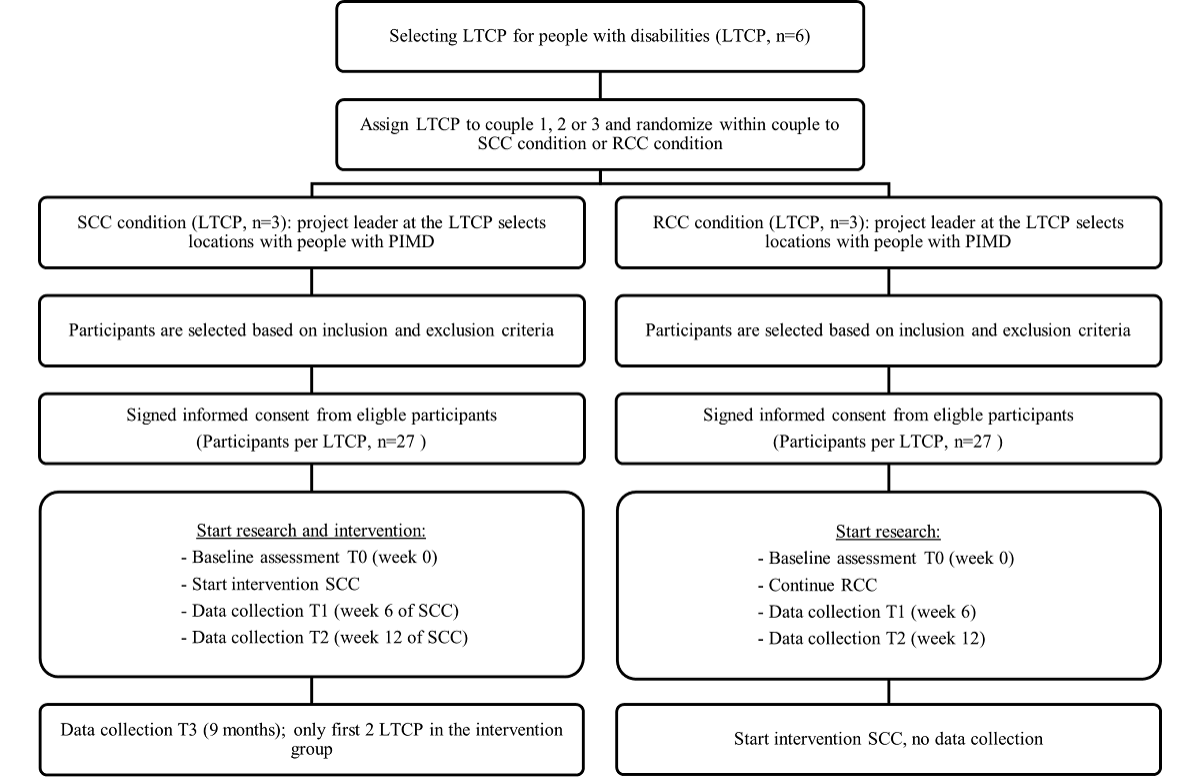
Flowchart of randomization, study inclusion, and measurements. LTCP: long-term care provider; PIMD: profound intellectual and multiple disabilities; RCC: regular continence care; SCC: smart continence care.

The use of SCC will set no limitations for concomitant care, such as the use of tranquilizers, diuretics, or laxatives. The caregivers propose participants at their locations based on the inclusion and exclusion criteria. The researchers, the product specialist of the supplier, behavioral therapist, and other experts within the long-term care provider are available for any consultation if there are any doubts about inclusion. People can be included even if they check 1 or 2 boxes of the soft exclusion criteria, as caregivers might see the added value of implementing SCC for such people. If people are eligible, their legal representatives receive an informational letter about the study and are asked to sign an informed consent form. The legal representatives are also offered to participate in the research. If they opt in by signing a second informed consent form, they will receive questionnaires about the person with PIMD. Figure 1 summarizes the recruitment and selection process.

### Intervention

#### Overview

In this study, SCC for people with PIMD will be provided by implementing Abena Nova with MediSens, produced by the Abena Group in collaboration with MediSens Wireless Inc [17]. This product was selected because it had higher market readiness than other products and was commonly used in Dutch pilots at the time of the grant application.

RCC consists of changing incontinence products, providing skin care if needed, and other hygienic activities, such as changing bed sheets and showering the person if a leakage has occurred. Most of the time, continence care is provided by following a fixed schedule, such as standard changes in the morning, around noon, and before going to bed (routines might vary among care teams). Leakage, specific behavior, or feces can cause deviations. Some people receive continence care during the night at fixed moments or when monitoring technology picks up noises during the night, indicating the need for care or continence care. In the RCC condition, the caregivers continue these regular routines.

**Figure 2:**
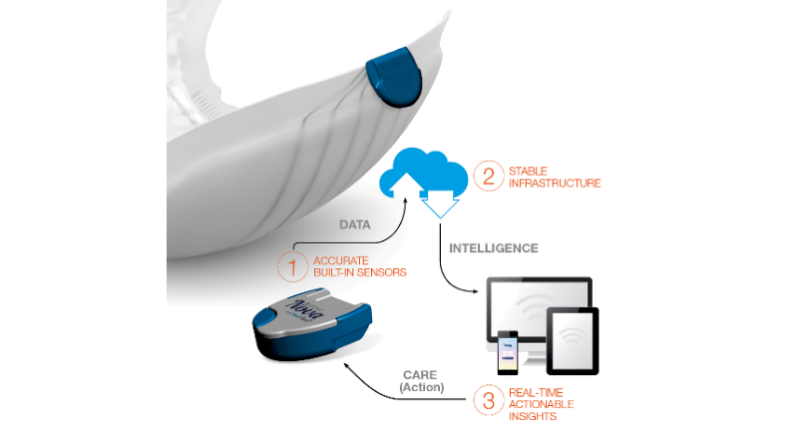
Schematic representation of the Abena Nova (reprinted with approval of Abena).

Abena Nova (Figure 2) consists of incontinence material with integrated sensors and a detachable and reusable clip. When the sensors become moist, the clip transmits this information to the receiver near the person with PIMD. The receiver sends this information to the cloud, and this information is displayed in an app called Wetsens (available on Android and iOS) and a web portal. When an adjustable threshold level for saturation is reached, the app sends a notification to inform the caregivers. Reaching the first threshold level triggers the notification “change desired” and displays an orange sign; then, caregivers have approximately 60 minutes to change the product. If the next threshold level is reached, a red sign is shown with the notification “risk of leakage.” Each receiver and app can monitor multiple users simultaneously, and each user can be monitored on several mobile devices at once. The sensor registers only urine. Feces is not detected and could even interfere with the registration of urine by blocking the sensors [27].

To provide SCC, caregivers need to be trained in using the technology and different incontinence materials. The supplier offers a 30-minute e-learning program and highly recommends that caregivers take this e-learning. Next, the team will have a live instruction (approximately 1 hour) from the supplier’s product specialist to discuss and practice using Abena Nova, such as how to attach the clip and use the app. It will be emphasized that perfectly fitting products (not only in size but also in applying it correctly) and reacting to notifications are essential to prevent leakages and provide SCC correctly.

For each participant, different teams providing continence care for 24 hours will be identified (residential care team, day care team, and night care team). Each team will have at least one ambassador who supports and motivates the team to provide SCC. This ambassador will receive additional training on using the web-based Wetsens portal. During the use of SCC, several meetings will be organized between the different ambassadors and product specialist of the supplier to discuss the individual participants and the results of SCC. If needed, alterations in SCC can be made to optimize the effectiveness, such as the size and absorption capacity of the material or the threshold level for notification. In addition, these meetings and the possibility of discussing the experiences will contribute to the adherence of caregivers to SCC.

Besides learning how to use the technology, caregivers will also need to change their working routines. Instead of having fixed schedules, notifications about “full incontinence products” should trigger continence care. Note that as feces is not detected by Abena Nova, normal schedule or observations apply for providing continence care in these cases. Altering work routines is difficult and time-consuming. Supporting and motivating the team will be needed and can be done by the product specialist of the supplier, ambassador, and project leader. The type and amount of support for each team might differ. Customization of the support for a team is recommended to increase adherence to SCC.

There are valid reasons for caregivers to discontinue SCC in individuals with PIMD, such as skin irritation or nonacceptance by the person with PIMD. Caregivers are free to make this choice by following what is best for the person with PIMD. The number of such discontinuations and their reasons are collected, as they hold valuable insights into the suitability of SCC. Data will still be collected for these persons according to the intention-to-treat principle.

#### Implementing SCC

Long-term care providers will receive implementation support through a practical step-by-step guideline (based on interviews with the supplier, users of SCC, the expertise of the research and implementation team [OS and VJCvC], scientific implementation protocol “Replicating Effective Programs” [28], and insights described in the work of Wensing and Grol [29] and implementation workbook by ZonMw [30]). This guideline is developed by the research team and will be updated during this project (Table 2). In this way, best practices and valuable insights into fostering or inhibiting factors for implementation can be transferred to the next organization. Meetings will be organized between the 6 project leaders of the participating organizations to exchange their best practices and difficulties. In addition, the project leaders will have weekly to monthly consultation sessions with members of the research team (VJCvC and OS). These moments of contact and the use of the guideline will be flexible according to the needs of the long-term care provider. This flexible approach to implementation is considered the most appropriate strategy for implementation and research projects in health care [31].

**Table 2:** A summary of implementation guidelines for the long-term care providers implementing smart continence care.

Phase	Examples of activities and focus
What to arrange beforehand	Involving relevant stakeholders (such as management, IT and facilities department, and internal health care services) in setting up the project team Assigning resources (time and finance) to the project Recruiting locations where people with profound intellectual and multiple disabilities live to implement smart continence care Indicating different care teams (24/7) involved in continence care for the people living at these locations Discussing vision on continence care with stakeholders Technical preparations (checking hardware, software, and support of IT department) Signing contract with the supplier
Preparing the teams and location	Making timeline per location and care team (training, technical installation, start smart continence care, and evaluations) Selecting people with profound intellectual and multiple disabilities who will receive smart continence care Completing training activities for caregivers to use smart continence care Technical preparations
Using smart continence care	On-site support for the care teams Continuous monitoring by the project leader on how the implementation (use of smart continence care) and changing work routines proceed; intervene if necessary
Decision-making on continuation and further uptake	End evaluation of the first use of smart continence care: Is it a success? When is it a success? What is needed to continue the uptake of smart continence care within long-term care facility? Decision-making with relevant stakeholders and plan of action for next steps Evaluating smart continence care with the different care teams involved, discussing specific cases, and using the Wetsens portal

#### Preparations Before the Research

We conducted a pilot study to test one of the research instruments, the continence diary, and learn about the implementation process. A total of 2 people with physical disability and moderate intellectual disability living at a long-term care facility used Abena Nova for 1 week. Three care teams were involved: the residential care, day care, and night care teams. The caregivers of the 2 participants were instructed on the use of SCC, research, and pilot’s goal. A think-out-loud session was conducted with 2 caregivers to discuss all research instruments and check their feasibility. A total of 2 caregivers and a project leader evaluated the implementation process and continence diary. This resulted in valuable insights for the practical step-by-step guideline and an improved continence diary by adding a clear-written instruction and better formulation of the questions.

### Measures

#### Overview

To assess the (cost)-effectiveness of SCC, outcome assessments will be performed at baseline (T0) and after 6 weeks (T1), 12 weeks (T2), and 9 months (T3). T3 only applies to the first 2 long-term care providers in the intervention group, as only these 2 care providers are assessed within the allotted time frame. Table 3 shows an overview of the questionnaire for each time point. Most data will be collected using paper and pencil, as this is easiest for caregivers. Research assistants will be trained for data entry, and the researchers will perform regular quality checks. Pseudonymized data will be stored at a secure site with limited access and separated from personal data such as names. Qualitative research will complement the effectiveness measures to evaluate the implementation. All data collection procedures and a data management plan (in Dutch) are available upon request from the corresponding author.

**Table 3:** Overview of the questionnaires over time.

			T0 (week 0)		Start SCC^a^		T1 (week 6)		T2 (week 12)		Start SCC		T3 (9 months)
**About the person with PIMD^b^**													
	Continence diary	✓				✓		✓				✓	
	Resource measurement questionnaire	✓				✓		✓				✓	
	EQ-5D-5L proxy 1^c^	✓						✓				✓	
	MIPQ^d^	✓						✓					
	QOL-PMD^e^	✓						✓					
	Goal Attainment Scale^f^							✓					
	General questionnaire	✓				✓		✓				✓	
**About the caregiver**													
	Work perception questionnaire—continence care	✓						✓					

^a^SCC: smart continence care, for the long-term care providers assigned to the SCC condition.

^b^PIMD: profound intellectual and multiple disabilities.

^c^The caregiver (the proxy) is asked to rate the patient’s health-related quality of life in their (the proxy’s) opinion.

^d^MIPQ: Mood Interest and Pleasure Questionnaire.

^e^QOL-PMD: Quality of Life of Persons With Profound Multiple Disabilities.

^f^Only applicable for the long-term care providers assigned to the SCC condition.

This study is an open-label trial, as it is not possible to blind the application of SCC to caregivers, participants, and families. Open-label trials are common among trials that investigate devices and other nonpharmaceutical interventions [32]. An independent outcome assessor unfamiliar with the treatment allocation will reduce the bias in the outcome assessment [33]. For this study, a statistician (WdH) will perform a blind assessment of the primary outcome measure.

#### Effectiveness Study

##### Primary Outcome

The number of leakages is registered in a “continence diary” for each participant for an entire week. Each caregiver providing continence care will enter the continence care provided per participant for an entire week in the printed diary. The primary outcome variable, whether leakage has occurred, will be registered by ticking a box. The diary will also hold information about the content of the incontinence material (ticking boxes for urine and feces separately). The data indicate the number of leakages per person per week at each time point. The change in the number of leakages over time within the intervention group will be compared with that in the group continuing their RCC.

##### Secondary Outcomes

###### Continence Care

Every instance of continence care is registered within the continence diary, providing information about the number of changes per participant per week. Shifts in the number of changes of incontinence material over time within one study arm will be compared with those in the other study arm. These registrations will include information about the reason for a change (eg, fixed schedule, notification generated by the sensor technology, behavior, leakage, or feces). Furthermore, the time spent on continence care and additional information on extra activities, such as skin care, washing the person, or changing bedsheets or clothing, will be registered.

###### Quality of Life

The number of instruments used to measure the quality of life of people with PIMD is limited. To select an instrument, it is important that there have been psychometric evaluations of the instrument within the target populations [34,35] and that the questionnaire be available in Dutch [36]. Two questionnaires developed explicitly for people with PIMD will be used to measure the effect on the multidimensional construct of quality of life [37-39] by comparing the difference in the scores of T2 to T0 between the 2 study arms. The “Mood, Interest, and Pleasure Questionnaire” is an indicator of subjective well-being [40]. This questionnaire consists of 22 items resulting in 3 subscales. The items are statements about the observed behavior of people with PIMD for the last 2 weeks. The proxies of the people with PIMD indicate their observations by scoring between 0 and 4 (0=never and 4=always occurred). The score is calculated by taking the sum of the corrected scores per subscale. A high score indicates high subjective well-being.

The questionnaire “Quality of Life of Persons With Profound Multiple Disabilities” [12] measures the objective quality of life and consists of 55 items resulting in 6 subscales. Each statement is scored between 0 and 2 (0=disagree, 1=partly agree, and 2=fully agree). The subscale score and total score are expressed as a percentage, between 0% and 100%. A score of 100% represents highly objective quality of life. Proxies can provide written clarifications to elaborate on their answers.

The proxies for both questionnaires are caregiver(s) of the person with PIMD and the legal representative (if opted in). Caregivers are allowed to consult with other colleagues when answering the questionnaires. Complementary interviews with caregivers will be held to explore how SCC influences the quality of life of persons with PIMD.

###### Setting Goals

During T0 and T2, an open question asks caregivers to describe the goal of providing SCC for the participant. In addition, the questionnaire at T0 asks the caregivers to elaborate on when this goal is met. The Goal Attainment Scale [41] depicts the extent to which this goal is met at T2 in the intervention groups. A total of 5 answer options are available to the respondents, ranging from “goal is not met, there has been a decline” to “the change exceeds the expectations, or more than just the goal is met.”

###### Perception of Work Related to Providing Continence Care

To measure a possible change in work perception, a web-based questionnaire is distributed among caregivers providing continence care to the participants. The web-based questionnaire is composed of and inspired by various questionnaires measuring different constructs related to the perception of work. First, the experienced burden of providing continence care is measured by 4 statements about work pressure (5-point Likert scale), scoring the physical and mental burden of continence care (scale: 0-10; 0=no burden and 10=very high burden). These items are inspired by Karasek [42] and Daems and Kunen [43]. Second, the construct of autonomy is measured by 4 items on a 5-point Likert scale specified for continence care, inspired by the Maastrichtse Autonomie Lijst (MAL) [44]. The overall satisfaction with continence care is measured by 1 item, which is scored between 0 and 10 (0=very low satisfaction and 10=very high satisfaction), and the participants are asked to explain the score. Work engagement is measured using the 9-item Utrecht Engagement Scale (UBES; a 7-point scale indicating frequencies) [45].

The web-based questionnaire at T2 contains 8 additional statements about SCC, which can be scored on a 5-point Likert scale (from totally disagree to totally agree). The RCC group selects the answer that best represents their opinion on the expectations of SCC. The SCC group selects the answer that best represents their actual opinion of SCC after using it for 12 weeks. Two additional open questions will explore the expected or experienced positive and negative effects of SCC.

Complementary in-depth interviews with caregivers will be held to explore their experiences with the implementation of SCC and its effect on their work routines. The interviews will be guided by a topic list available upon request from the corresponding author.

#### Economic Evaluation

##### Overview

The economic evaluation will use a trial-based approach and will be performed from a societal perspective, as recommended by the Dutch guidelines for cost calculations in health care [46]. Besides the societal perspective, it is relevant to adopt the perspective of the long-term care providers, as they themselves make the primary decision regarding whether to implement SCC. The trial-based economic evaluations will include both a cost-effectiveness analysis (CEA) and cost utility analysis (CUA). The time frame of the study is 9 months. Within the RCC group, data will be collected at T0, T1, and T2; there will be no data collection at T3, but the measurement of T2 will serve as a proxy for T3 in the RCC condition. This can be done because the target population is expected to be stable, and having additional measures will cause an unnecessary burden to the caregivers filling in these questionnaires. Besides, this gives long-term care providers randomized in the RCC condition the opportunity to start the implementation of SCC immediately after T2. Within the SCC group, data will be collected at T0, T1, T2, and, for the first 2 intervention groups, at T3. This follow-up measurement will provide data about whether and to what extent the effect remained and whether medical costs changed for the participants who received SCC. The cost prices will be expressed in euros based on the cost prices in 2022. If necessary, the existing cost prices will be updated to those in 2022 using the consumer price index available from Statistics Netherlands [47]. In this economic evaluation, discounting is irrelevant, as the follow-up period is less than a year.

##### Estimation of Costs

The cost within health care and cost for participants and their families will be taken into account (Table 4 presents an overview). To identify relevant cost aspects, we have adopted an iterative process, similar but more condensed, as described by Thorn et al [48]. A search performed in the DIRUM (Database for Instruments of Resource Use Measurements; June 2021) did not provide instruments that could serve as a basis for resource use collection. Therefore, to determine the cost aspects of each category, previous cost studies on SCC [18,19,49] and field observations provide an initial list of cost aspects directly related to continence care. This list is finalized by a brainstorming session with different employees within a long-term care facility (people with and without experience in using SCC, such as coordinator night care, physiotherapist, and project leader). These cost aspects together with the resource measurement questionnaire, using a selection of relevant items from the *i*MCQ (*i*MTA Medical Cost Consumption) [50], are used to indicate all possible impacts on health care costs. Table 4 provides a general overview of the cost aspects, which instruments will be used to estimate resource use, and which unit prices will be used to valuate the resource use to calculate the costs. It also includes some cost aspects that are not further investigated because of the overall research costs (time-consuming and complicated to measure and validate) and the expected negligible impact on the total cost [51]. Costs within other sectors, such as productivity loss or absenteeism in a work-related setting, are not relevant to people with PIMD because they are unable to do (voluntary) work owing to their disability.

**Table 4:** Unit costs and how these are measured and valued.

				Method to measure resource use		How it is valued (source of unit cost)		Remarks and examples
**Cost within health care**								
		Health care costs	Continence diary and resource measurement questionnaire		Dutch reference prices		Staff costs of continence care and all health care resources used, including medication	
		Related costs	Estimation based on continence diary		Dutch reference prices		Laundry and waste disposal costs	
**Intervention costs**								
		Material costs (disposable)	Continence diary		Market price		Incontinence material, gloves, bathing gloves, and skincare	
		Material costs (reusable)	Long-term care provider and supplier		Market price		Only applicable for the intervention group; clips, receivers, and care phones (optional)	
		Licensing fee for smart continence care	Number of days per user		Market price		Only applicable for the intervention group	
		Education and instructions to caregivers on the intervention	Information from the project leader at the long-term care provider		Dutch reference prices		Use Dutch guideline for costing to calculate	
		Costs of ICT^a^ for the implementation and facilitation of the intervention within the long-term care provider	Information from the project leader at long-term care provider		Salary ICT		Use Dutch guideline for costing to calculate	
		Costs of project managing the implementation and facilitation of the intervention within the long-term care provider	Information from the project leader at long-term care provider		Dutch reference prices		Use Dutch guideline for costing to calculate	
**Cost for the participants and their families**								
		Costs of nonvisit because of continence leakage	Expert opinion		Not valued in monetary terms		Expert opinion is needed to estimate the impact	
		Costs of shorter visits because of leakage	Expert opinion		Not valued in monetary terms		Expert opinion is needed to estimate the impact	
		Costs of change in leisure activities	General questionnaire and expert opinion		Not valued in monetary terms		Expert opinion is needed to estimate the impact	

^a^ICT: information and communication technology.

##### Estimation of the Effects

For the CEA, the change (T3-T0) in the number of leakages will be used as the outcome measure. Within the RCC group, the number of leakages at T2 will serve as a proxy for T3, still representing the RCC condition. Information regarding this outcome will be collected through the continence diaries.

For the CUA, the quality-adjusted life years (QALYs) will be used as the study outcome. To calculate these QALYs, the proxy 1 version of the EQ-5D-5L will be used because a person with PIMD cannot self-report. This instrument is seen as a valid, reliable, and more discriminating measurement compared with the previous 3-level version (EQ-5D-3L) and is often used in CUAs [52,53]. The primary caregiver acts as the proxy, and if opted in, the legal representative is the second proxy. The questionnaire consists of two parts: the EQ-5D, which is a descriptive system providing a health state, and a visual analog scale. The health state provided by EQ-5D consists of five dimensions (mobility, self-care, usual activities, pain/discomfort, and anxiety/depression), each scored on a 5-point scale, giving 3125 possible health states [54]. These health states are valued using the Dutch utility score [52]. The visual analog scale is scored between 0 and 100, with a lower score indicating a lower health-related quality of life.

#### Process Evaluation

The difficulties and milestones of the implementation process will be discussed during regular meetings between the project leader of the long-term care provider and the implementation specialist and researcher (OS and VJCvC). Field notes and observations of these meetings and other important events (such as training sessions, evaluations, or site visits) will be taken by the researcher (VJCvC). In addition, interviews will be held with project leaders after implementing SCC using a topic list as an interview guideline. The field notes can be used to ask additional questions about certain events. Furthermore, the caregivers will be interviewed about their experiences with implementing and using SCC. Purposive sampling will be used to select caregivers for the interviews, aiming to obtain a wide variety of experiences from different long-term care providers and contributing to the credibility of the findings [55]. The field observations will guide this purposive sampling. All participants will be asked for their consent to audio record the interviews.

### Analysis

#### Effectiveness Study

Although it is not possible to blind the intervention during the trial, statistical analyses of the primary outcome measure will be performed by a statistician (WdH) unfamiliar with the treatment allocation. The data set will contain dichotomous variables (using condition “A” or “B”), referring to either SCC or RCC. The meaning of this dichotomous variable is randomly assigned using random.org. Primary outcome data will be analyzed using a generalized linear mixed model with a Poisson link function (R version 4.0+, package lme4) containing 3 levels: measurements within participants nested within long-term care providers. Time will be coded into binary dummy variables and added to the model as an interaction with the intervention, allowing for testing the difference between the treatment arms at each time point. Secondary outcome data will be analyzed similarly, albeit with the appropriate link function matching the type of outcome data. While mixed models allow missing data and hence appropriately serve the intention-to-treat principle, completer analyses will also be performed. The results will be described in accordance with the CONSORT (Consolidated Standards of Reporting Trials) guidelines for randomized controlled trials [56,57]. This study will be reported by following the CHEERS (Consolidated Health Economic Evaluation Reporting Standards) 2022 guidelines for reporting economic evaluations in health care [58].

#### Economic Evaluation

The sample size for the economic evaluation is based on the power analysis performed for the CRT. The primary (base case) analyses will be performed according to the intention-to-treat principle. This means that data from all the participants will be used, regardless of whether they received the intervention. For the analyses, we will use SPSS (version 28) statistical software and Microsoft Excel (Office 365; version 2205). However, if correction for baseline differences is needed, R (version 4.0+, package Ime4) will be used. Missing measurements will be handled using multiple imputation. Before the start of the analyses, a baseline analysis will be performed to examine the comparability of the groups at baseline for both costs and outcomes. If necessary, methods will be applied to control for differences in baseline measurements [59,60].

Owing to the expected skewness of cost data, besides means (and SDs), medians and IQR will be presented. The incremental cost-effectiveness ratios (ICERs) will be calculated for both CEA and CUA. The ICER will be calculated as follows: ICER = (Ci − Cc)/(Ei − Ec), where Ci is the total cost of the new intervention (SCC), Cc is the total cost of the comparator (RCC), Ei is the effect at the 9-month follow-up for SCC, and Ec is the effect at the 12-week follow-up for RCC, which is expected to be a good predictor for the situation after 9 months of not having the intervention. The robustness of the ICER will be checked by nonparametric bootstrapping. Bootstrap simulations will also be conducted to quantify the uncertainty around the ICER, yielding information about the joint distribution of cost and effect differences. Bootstrap replications will be used to calculate 95% CIs around the costs based on the 2.5 and 97.5 percentiles. The bootstrapped cost-effectiveness ratios will be plotted in a cost-effectiveness plane, in which the vertical axis will reflect the difference in costs, and the horizontal axis will reflect the difference in effectiveness. In addition, to demonstrate the robustness of our base-case findings, various sensitivity analyses will be performed, such as subgroup analyses examining the effect at 3 months. In these analyses, assumptions made in the base-case analysis will be varied to investigate their possible influence on the study outcomes, for example, by varying the cost prices and volumes between minimum and maximum.

The choice of treatment depends on the maximum amount of money that the society is prepared to pay for a gain in effectiveness, which is called the ceiling ratio. Therefore, the bootstrapped ICERs will also be depicted in a cost-effectiveness acceptability curve, showing the probability that the intervention is cost-effective, using a range of ceiling ratios. The ceiling ratio for the societal cost per QALY depends on the disease burden. Severe motor and cognitive impairments result in a disease burden of 0.425 (95% uncertainty interval 0.286-0.587) [61]. In the Netherlands, the disease burden is currently estimated to be €50,000 (US $48,847.92, currency rate as per October 19, 2022) per QALY (disease burden of 0.41-0.70) [62].

#### Process Evaluation

The interviews will be audio recorded and summarized by one of the researchers. The project leaders who participate are offered a member check on this summary to check the researcher’s understanding of what is said and meant by the participant [55]. A second researcher will review these summaries by listening to the audio recordings and adding illustrative quotes. Using the software program Atlas.ti (version 9.1), these summaries will be coded through an iterative process. In this process, several researchers will be involved in discussing data analysis and increasing the credibility of the findings.

## Results

This project received approval for funding on December 5, 2019, by ZonMw, the Netherlands Organization for Health Research and Development (grant 80-85300-98-19110). The funding period is 36 months. Owing to the COVID-19 pandemic, we received an extension of 6 months.

Data collection from the 160 participants living in one of the 6 long-term care facilities and their caregivers will provide insights into the effectiveness, cost-effectiveness, and implementation process of SCC compared with RCC. As of June 2022, we enrolled 118 of the 160 participants. The enrollment of participants will continue in Q3 and Q4 of 2022.

## Discussion

### Expected Findings

We expected that SCC would decrease the number of leakages compared with RCC when used for people with PIMD. A decreasing number of leakages and the avoidance of unnecessary changes are expected to have a positive effect on the quality of life of people with PIMD. Because disruptive activities such as changing clothing and showering owing to leakages and skin irritation due to long exposure to wet incontinence material are expected to decrease, this will result in less agitated behavior. More personalized continence care is also expected to be cost-effective. Despite the higher cost of the material and cost of implementing SCC, it has the potential to save time for caregivers and decrease the use of products such as skin care products. In addition, the evaluation of the implementation process will provide valuable insights for long-term care providers. One of the insights expected is that facilitating employees with time and resources for implementation is important, as is setting up a project team and the early involvement of relevant stakeholders.

### Strengths and Limitations

Although research guidelines argue that to measure the effectiveness of an intervention, the variation between individuals delivering this intervention should be minimized [20], providing continence care to people with PIMD is done by a wide variety of caregivers. Successful implementation of SCC and thus its observed effectiveness depends on the caregivers using it and the degree to which they change their work routines. This research will be conducted in a real-life setting, in which a great variety of other factors might influence the outcomes. Therefore, this pragmatic CRT (effectiveness study) is less controlled than expected from a controlled trial.

Furthermore, caregivers may alter their behavior and compliance with (smart) continence care because they are aware of the ongoing research (Hawthorne effect). However, we argue that this real-life research will provide more valuable insights into the added value that SCC can bring to long-term care facilities, caregivers, and, most importantly, people with PIMD [63]. Furthermore, including 6 different long-term care providers from across the Netherlands increases generalizability. Adherence to the CONSORT [57] and CHEERS 2022 [58] guidelines increases the quality of data reporting.

To incorporate the voice of the people with PIMD and the effect of the intervention on their quality of life, we must use proxies because the researchers lack the experience to understand the participants’ behaviors and translate them into meaningful outcomes. By inviting caregivers and relatives to act as a proxy, we aim to incorporate the experience of people with PIMD in the best possible way. This could be seen as a limitation, as the experiences are never firsthand; however, receiving firsthand answers from people with PIMD is not possible.

Overall, this study protocol presents a unique (cost)-effectiveness research for a population rarely researched in such a way, people with PIMD. To the best of our knowledge, there has not been a large-scale study of this size (N=160) within this population before. This study investigates the effectiveness, cost-effectiveness, and cost utility of SCC compared with RCC. Thorough research on the effects, added value, and costs within this real-life setting will help funding bodies and policy makers at long-term care facilities with important information to make an informed decision about whether to implement smart continence products for people with PIMD. This is relevant because SCC enables person-centered care, which is an important goal, as stated by the Health and Youth Care Inspectorate.

### Dissemination Plan

This study will produce several result papers, which will be submitted to scientific journals. Furthermore, this study is part of the dissertation of the first author (VJCvC), meaning that all the results will be available to the public through the expected dissertation. In addition, all long-term care providers and legal representatives, if interested, will receive a public-friendly summary of the results.

## Appendix

Multimedia Appendix 1 The World Health Organization trial registration data set.

## Abbreviations

CEA: cost-effectiveness analysis
CHEERS: Consolidated Health Economic Evaluation Reporting Standards
CONSORT: Consolidated Standards of Reporting Trials
CRT: cluster randomized trial
CUA: cost utility analysis
DIRUM: Database for Instruments of Resource Use Measurements
ICC: intraclass correlation coefficient
ICER: incremental cost-effectiveness ratio
iMCQ: iMTA Medical Cost Consumption
IRR: incidence rate ratio
MAL: Maastrichtse Autonomie Lijst
PIMD: profound intellectual and multiple disabilities
QALY: quality-adjusted life year
RCC: regular continence care
SCC: smart continence care
SPIRIT: Standard Protocol Items: Recommendations for Interventional Trials
UBES: Utrecht Engagement Scale

## Data Availability

The data sets generated during or analyzed during this study are not publicly available because of the vulnerable position of the participants but are available from the corresponding author upon reasonable request.
