# Supplementary material for: Smart Continence Care for People With Profound Intellectual and Multiple Disabilities: Protocol for a Cluster Randomized Trial and Trial-Based Economic Evaluation
Source: JMIR Res Protoc. 2022 Nov 22;11(11):e42555. doi: 10.2196/42555 (PMC9727688; doi:10.2196/42555)
Supplement: Multimedia Appendix 1 [file resprot_v11i11e42555_app1.docx]

**Table S1.** The World Health Organization trial registration data set.

| Data category | Information |
| --- | --- |
| Primary registry and trial identifying number | - ClinicalTrials.gov identifier: NCT05481840 |
| Date of registration in primary registry | - August 1, 2022 |
| Secondary identifying numbers | - N/A^a^ |
| Sources of monetary or material support | - ZonMw Programma Goed Gebruik Hulpmiddelen |
| Primary sponsor | - ZonMw Programma Goed Gebruik Hulpmiddelen |
| Secondary sponsors | - Academische Werkplaats Sterker op Eigen Benen Radboudumc (in kind) - Abena Healthcare |
| Contact for public queries | - Brigitte Boon, principal researcher - Phone: 0031 88-3779999 - Email: Brigitte.Boon@academyhetdorp.nl - Organization: Academy Het Dorp, Arnhem, the Netherlands |
| Contact for scientific queries | - Marieke Gielissen, coprincipal researcher - Phone: 0031 88-3779999 - Email: Marieke.Gielissen@academyhetdorp.nl - Academy Het Dorp, Arnhem, the Netherlands |
| Public title | - Smart Diaper implementation and research project |
| Scientific title | - Smart Continence Care for People with Profound Intellectual and Multiple Disabilities: Protocol for a Cluster Randomized Trial and Trial-Based Economic Evaluation |
| Countries of recruitment | - The Netherlands |
| Health conditions or problems studied | - People with profound intellectual and multiple disabilities using incontinence products |
| Interventions | - Intervention: smart continence care (Abena Nova)  Comparator: regular continence care (care as usual) |
| Key inclusion and exclusion criteria | - Ages eligible for the study: ≥18 years - Sexes eligible for the study: both - Accepts healthy volunteers: no - Inclusion criteria: adult participant (aged ≥ 18 years), living within a long-term care facility, having profound intellectual and multiple disabilities, and using incontinence products - Exclusion criteria: pica disorder and using a permanent catheter |
| Study type | - Interventional - Allocation: a cluster randomized 1:1 - Intervention model: cluster randomized trial, pragmatically rolled out in 3 waves - Masking: none, yet outcomes assessor will be blinded - Primary purpose: care |
| Date of the first enrollment | - August 2021 |
| Target sample size | - 160 |
| Recruitment status | - Recruiting |
| Primary outcome | - Number of leakages |
| Key secondary outcomes | - Quality of life of a person with profound intellectual and multiple disabilities - Work perception of caregivers - Cost-effectiveness - Cost utility |

^a^N/A: not applicable.
